# Pediatric Ectopic Cushing Syndrome Caused by Hepatic Neoplasms: A Case Report and Systematic Review

**DOI:** 10.7759/cureus.36852

**Published:** 2023-03-29

**Authors:** Ganesh Jevalikar, Shruthi Ravindra, Pavan Kumar Reddy, Sagar Reddy S L, Vijaya Sarathi

**Affiliations:** 1 Pediatric Endocrinology, Max Super Speciality Hospital, New Delhi, IND; 2 Endocrinology, Diabetes and Metabolism, Narayana Medical College, Nellore, IND; 3 Endocrinology, Diabetes and Metabolism, Sai Balaji Diabetes and Endocrine Center, Proddatur, IND; 4 Endocrinology, Diabetes and Metabolism, Vydehi Institute of Medical Sciences and Research Centre, Bengaluru, IND

**Keywords:** venous adrenocorticotropic hormone sampling, preoperative adrenocorticotropic hormone immunohistochemistry, calcifying nested stromal epithelial cell tumor, primary hepatic neuroendocrine tumor, ectopic cushing’s syndrome

## Abstract

Ectopic adrenocorticotropic hormone (ACTH) syndrome (EAS) is rare in children, and localizing the source of EAS is often challenging. Here, we report EAS in an adolescent boy who presented with Cushingoid features and had endogenous ACTH-dependent hypercortisolism on hormonal evaluation. Abdominal ultrasound and CT revealed a hepatic lesion with characteristics suggestive of hemangioma, whereas the lesion was tracer non-avid on ^68^Ga-DOTANOC positron emission tomography/CT. A regional sampling of ACTH was done to confirm the hepatic lesion as the source of EAS, and a definitive ACTH gradient was observed between the hepatic vein and the right internal jugular vein. Further, a preoperative biopsy of the lesion revealed a small round cell tumor with positive immunostaining for ACTH and synaptophysin, suggestive of a neuroendocrine tumor. The patient was managed with partial hepatectomy, resulting in hormonal and clinical remission of Cushing syndrome. In a systematic review of pediatric EAS due to primary hepatic tumors (n = 11), calcifying nested stromal epithelial cell tumors were the most common. EAS-associated hepatic tumors were larger (≥10 cm) except benign primary hepatic neuroendocrine tumors (PHNET). The latter were misdiagnosed as hemangioma in two cases by anatomical imaging but correctly diagnosed by somatostatin receptor scintigraphy. Hepatic tumors causing EAS in children required extensive resection, except benign PHNET. Nevertheless, all benign tumors with an uncomplicated perioperative course demonstrated disease-free survival over a median follow-up period of two years.

## Introduction

Endogenous Cushing syndrome (CS) is rare in childhood and adolescence. Endogenous CS is either adrenocorticotropic hormone (ACTH)-independent (primary adrenal etiology) or ACTH-dependent. The most common ACTH-dependent CS is excess ACTH production from a pituitary corticotroph adenoma (Cushing disease, CD) but rarely may also result from ectopic ACTH syndrome (EAS) due to excess production of ACTH and/or corticotropin-releasing hormone (CRH) from various tumors outside the pituitary gland (10%). Localizing the source of ACTH-dependent CS is often challenging, especially in children. Frequent negative localization of pituitary microcorticotropinomas, the most common cause of CD, by MRI, unavailability of CRH, limited availability of bilateral inferior petrosal sinus sampling (BIPSS) in India, and inaccuracy of high-dose dexamethasone test (HDDST) pose challenges to distinguish CD from EAS. Even when a diagnosis of EAS is inferred from the aforementioned tests, localizing the source of EAS is a further diagnostic challenge. CT and MRI are the most commonly used initial modalities for localizing the source in EAS but may fail in approximately 33-44% of cases. A higher sensitivity (80-90%) has been reported for somatostatin receptor scintigraphy (SRS) [1]. Here, we report the case of a child in whom these conventional modalities failed to confirm the source of EAS that was ascertained with novel diagnostic modalities. In addition, we have systematically reviewed the pediatric EAS patients reported in the literature.

## Case presentation

**Figure 1:**
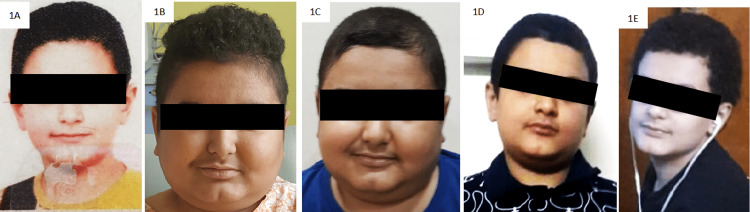
Clinical photographs of the patient six years before the presentation (1A), at diagnosis (1B), and one month (1C), four months (1D), and one year (1E) after excision of the hepatic neuroendocrine tumor.

A 10.5-year-old Iraqi boy presented with excessive weight gain (10 kg over one year) and headache. The patient also complained of rounding of the face, thinning of scalp hair, dark-pink stretch marks, and acne. There was no history of exogenous glucocorticoid intake, hyperpigmentation, or visual disturbances. He was detected to have hypertension, which was treated with multiple anti-hypertensives. He was born of a non-consanguineous marriage and had an uneventful birth history. On examination, weight was 49.6 kg (97th centile), height was 135.5 cm (25th centile), and body mass index was 27 kg/m^2^ (>97th centile). His blood pressure was 120/94 mmHg (>95th centile). The child had moon facies; truncal adiposity with relatively thin extremities; wide, purple striae over the abdomen and lower limbs; papular acne; facial plethora; thinning of scalp hair; and seborrhea (Figure 1).

Mild proximal muscle weakness (grade 4/5) was elicited on examination. He had pubic hair stage 2 with stretched penile length of 5 cm and bilateral testicular volume of 2 mL each. The rest of the systemic examination was unremarkable. The biochemical evaluation revealed low-normal potassium (3.6 mEq/L) and normal liver and renal function tests. The hormonal evaluation confirmed endogenous CS (Table 1) that was ACTH-dependent.

**Table 1:** Results of biochemical evaluation at the baseline visit and after ketoconazole treatment. *: Tests are done after stopping ketoconazole for 72 hours. LDDST: low-dose dexamethasone suppression test; HDDST: high-dose dexamethasone suppression test

Parameter (reference range)		Baseline visit	One year on medical treatment*
Serum cortisol (5-22 µg/dL)	8 AM	47.0	76.0
(<1.8 µg/dL)	LDDST	86.0	-
(<1.8 µg/dL)	Overnight HDDST	86.0	-
24-hour urine-free cortisol (28.5–213.7 µg)		1,597.83	495.65
Adrenocorticotrophic hormone (10–60 pg/mL)	8:00 AM	28.18	73.64
Serum potassium (3.5–5.5 mEq/L)		3.6	3.0
Alanine transaminase/Aspartate transaminase (7–56 IU/L)		171/44	68/33
Fasting plasma glucose (70–99 mg/dL)		108	112

**Figure 2:**
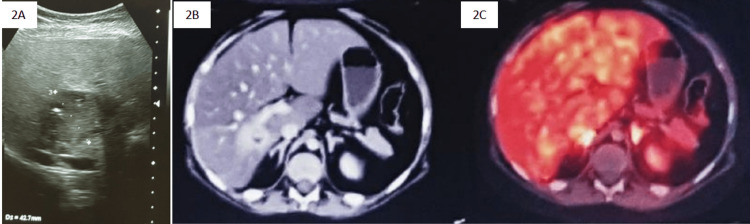
Ultrasonogram showing mixed echogenic lesion measuring 54 × 43 × 41 mm with internal cystic areas and calcification (2A); 68Ga-DOTANOC PET/CT showing an ill-defined enhancing mass measuring 41 × 30 mm in segment VII and VI of the liver with a small central area of hypodensity, preserved perilesional fat, and persistent enhancement on delayed phase (2B) that was DOTANOC non-avid (2C) suggestive of hemangioma.

Dynamic contrast MRI of the pituitary was unremarkable whereas CT of the abdomen revealed a 41 × 30 mm lesion in the liver with enhancement in the arterial phase (HU: 102) that persisted in the delayed phase (HU: 103) suggestive of hemangioma (type II dynamic enhancement curve). To further delineate this lesion, ^68^Ga-DOTANOC positron emission tomography (PET)/CT was done, which revealed a somatostatin non-avid lesion (Figure 2B, 2C). This finding further reduced the probability of the lesion being the source of EAS. As the patient desired to return to the native country, further evaluation could not be done, and the patient was started on oral ketoconazole 400 mg/day which was further increased to 800 mg/day. Although ketoconazole achieved a partial reduction of urinary free cortisol, there was a clinical progression of CS in the form of further weight gain (5.4 kg), poor linear growth (2.8 cm/year), uncontrolled hypertension, and worsening of extremity thinning, striae, and muscle weakness. The repeat biochemical evaluation showed persistent hypercortisolemia with hypokalemia and impaired fasting glucose (111 mg/dL). He underwent repeat imaging of the pituitary and the abdomen. While the MRI of the pituitary was unremarkable, the ultrasound of the abdomen (Figure 2A) revealed an increase in the size of the hepatic lesion to 54 × 43 × 41 mm with internal cystic areas and calcification and minimal internal vascularity.

**Figure 3:**
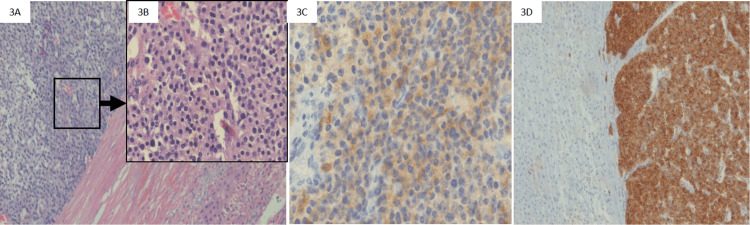
Section shows fibrofatty tissue (3A) infiltrated by a small round cell tumor in a vague acinar pattern and cords (3B). Immunohistochemistry was positive for adrenocorticotropic hormone (3C) and synaptophysin (3D).

A regional sampling of ACTH was done to confirm this lesion as the source of ACTH secretion, and a definitive ACTH gradient was observed between the hepatic vein (ACTH: 92 pg/mL) and the right jugular vein (ACTH: 25 pg/mL). Further, the biopsy from the lesion suggested a small round cell tumor with positive immunostaining for ACTH and synaptophysin (Figures 3A-3D) suggestive of a neuroendocrine tumor (NET). The serum level of chromogranin-A was elevated (401 ng/mL, normal: <76.3). The patient underwent right hepatic vein sparing and right posterior partial hepatectomy consisting of segments VI and VII. Postoperatively, remission of CS was confirmed by normalization of blood pressure, hyperglycemia, and hypokalemia along with low morning serum cortisol level (2 µg/dL) on the 10th postoperative day. The patient was initiated on hydrocortisone replacement (10 mg/m^2^/day) and monitored every three months with morning serum cortisol. A year later, morning serum cortisol had normalized (7.9 µg/dL) with ACTH-stimulated cortisol of 21.2 µg/dL; hence, glucocorticoid replacement was stopped. A height gain of 12 cm was documented in the first postoperative year. At his last follow-up after five years of surgery, he was disease free.

## Discussion

Systematic review

**Figure 4:**
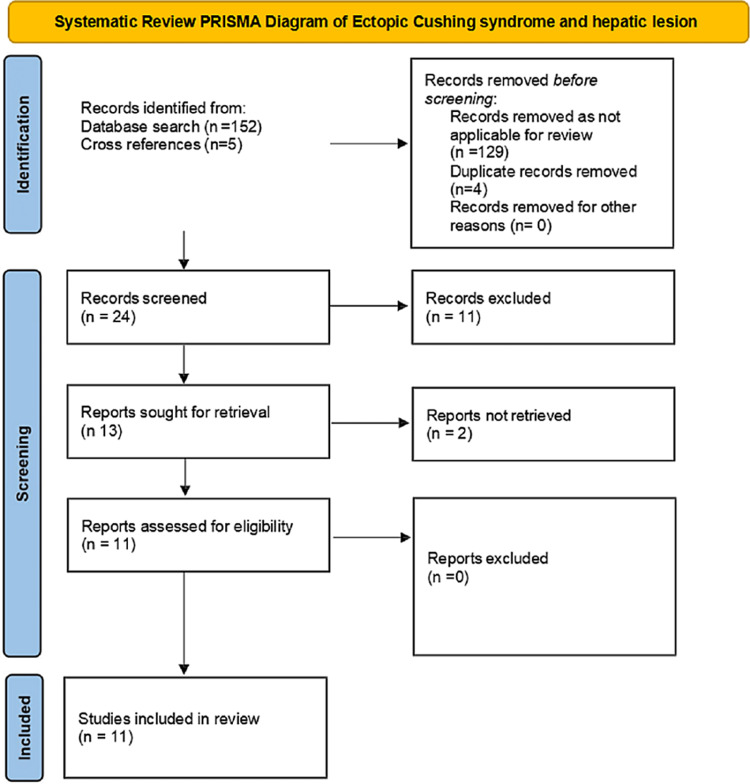
PRISMA flow chart for selection of articles for the systematic review. PRISMA: Preferred Reporting Items for Systematic Reviews and Meta-Analyses

A systematic review of the literature was performed following Preferred Reporting Items for Systematic Review and Meta-Analyses (PRISMA) guidelines. The PubMed database was searched in November 2022 with search terms “ectopic Cushing” AND “liver” for articles published between January 2000 and October 2022. The search yielded 152 articles; in addition, five articles were identified from cross-referencing. Of these, a total of 24 articles were screened, 13 were sought for retrieval, and 11 were included in the final analysis (Figure 4).

The systematic review consisted of 11 children and adolescents with EAS due to calcifying nested stromal epithelial cell tumor (CNSET, n = 7), PHNET (n = 3), and hepatoblastoma (n = 1) (Table 2) [2-11]. One PHNET was metastatic at presentation. Cushingoid features were universal with a median duration of symptoms of 2.5 months (range: 0.25-6 months, n = 6). The median tumor size was 12 cm (2.5-19.5 cm, n = 7), with most (n = 6) being ≥10 cm. All CNSETs were ≥10 cm, whereas, of the two benign PHNETs, one was 2.5 cm, and the size was not mentioned for the other but apparently smaller. The median morning serum cortisol was 59.1 µg/dL (23.9-119.6 µg/dL, n = 8), whereas plasma morning ACTH was 174.5 pg/mL (57.5-945.4 pg/mL, n = 9). All anatomical imaging modalities (ultrasound, CT, and MRI) identified the hepatic lesions, although the two benign PHNET were misdiagnosed as hemangioma. The latter two lesions were diagnosed by SRS. Notably, the two patients with CNSET who underwent SRS had no tracer uptake in the lesion. All patients were managed surgically by variable degrees of hepatectomy (tri-segmentectomy to hepatectomy), with additional Whipple’s procedure and orthotopic transplantation in one each. Adrenostatic drugs (ketoconazole/metyrapone) and chemotherapy (adjuvant/neoadjuvant) were used in three and two patients, respectively. All tested patients had positive immunostaining for ACTH (n = 8) and CRH (n = 3). Over a median follow-up duration of two years (range: 10 days to 2 decades, n = 11), two patients died; one in the postoperative period due to sepsis (10 days), and the other due to progressive metastatic PHNET (eight months).

**Table 2:** A systematic review of pediatric EAS patients with hepatic neoplasm. *: Initially misdiagnosed as hemangioma. ACTH: adrenocorticotropic hormone; BWS: Beckwith-Weidemann syndrome; CK19: cytokeratin 19; CNSET: calcifying nested stromal epithelial tumor; CRH: corticotropin-releasing hormone; CT: computed tomography; DF: disease-free; DM: diabetes mellitus; F: Female; FDG: 18F-flurodeoxy glucose positron emission tomography/computed tomography; HG: hemangioma; HTN: hypertension; KC: ketoconazole; MRI: magnetic resonance imaging; M: male; MTP: metyrapone; PHNET: primary hepatic neuroendocrine tumor; RBC: red blood cell-labeled scintigraphy; SRS: somatostatin receptor scintigraphy (111In-pentetreotide or unspecified); Sym Dur: symptom duration; USG: ultrasonogram

Serial number	Author, year [reference]	Age (Months)	Gender	Tumor	Clinical features	Sym Dur (Months)	Tumor size (cm)	Morning cortisol (µg/dL)	24-hour UFC (µg)	Morning ACTH (pg/mL)	Imaging	Treatment	IHC	Outcome
1	Heerema-McKenney et al., 2009 [2]	144	F	CNSET	Cushingoid, abdominal mass	--	--	--	--		CT+	Right hepatic lobectomy	--	2 years DF
2	Heerema-McKenney et al., 2009 [2]	132	F	CNSET	Cushingoid, abdominal mass	--	12	--	--	545.45	--	Left hepatectomy	ACTH	2 years DF
3	Rod et al., 2009 [3]	204	F	CNSET	Facial edema, palpable mass	--	13.2 × 9.2	23.92	1264	113.64,	USG+, CT+, SRS-	Left hepatectomy	ACTH, CRH	2 months DF
4	Grunewald et al., 2010 [4]	81	F	Hepato blastoma	Cushingoid, DM, HTN, abdominal mass	2	1,000 mL	67.35	1,289	184–819	USG+, CT+	Excision and adjuvant polychemotherapy	ACTH, weak CRH,	2 decades DF
5	More et al., 2011 [5]	204	F	CNSET	--	--	--	--	--	--	CT+, FDG+, SRS-	Excision		4 years, DF
6	Geramizadeh et al., 2012 [6]	96	M	CNSET	Cushingoid, HTN, DM	3	10 × 9	63.43	398	945.45	CT+	Hepatectomy	ACTH	Died, sepsis
7	Luca et al., 2013 [7]	108	M	PHNET*	Cushingoid, DM, HTN	3	2.5	52.27	3,622	174.55	USG, CT, MRI: HG SRS+, FDG-, RBC-	MTP + wedge liver resection	ACTH	8 months, DF
8	El Zein et al., 2014 [8]	108	M	PHNET*	Cushingoid	6	--	119.62		100	USG, CT, MRI: HG SRS+, FDG-, RBC-	Excision	ACTH	14 months, DF
9	Karageorgiadis et al., 2015 [9]	165	F	Metastatic PHNET	Cushingoid	--	12	59.8	793	57.5	CT+, MRI+, FDG+	Whipple’s procedure	ACTH CRH	Died after 8 months
10	Weeda et al., 2016 [10]	192	M	CNSET	Cushingoid	2	19.5	28.45		285	MRI+	KC + tri-segmentectomy	ACTH	13 years, DF
11	Tehseen et al., 2017 [11]	156	F	CNSET BWS	Cushingoid, HTN, abdominal mass	0.25	10.3 × 15.9 × 17.3	58.4		170	CT+	Neoadjuvant chemo + KC + orthotopic liver transplantation	--	24 months, DF

Here, we report a child with ACTH-dependent CS and several challenges to localize the source of excess ACTH. A negative pituitary MRI suggested either missed microcorticotropinoma or EAS. An unsuppressed HDDST favored EAS whereas CRH-stimulation test or CRH-stimulated BIPSS could not be considered due to the unavailability of CRH in India and, hence, further evaluated with imaging of the chest and abdomen to localize the possible source of EAS [12]. The common causes of EAS in children aged more than eight years include bronchial, thymic, and pancreatic NETs [9], whereas the imaging revealed a possible source of excess ACTH in the liver of our patient. The reported causes of liver tumors associated with pediatric EAS include CNSET, PHNET, or hepatoblastoma. Although not reported in children, hepatic metastases from a primary NET elsewhere can also be associated with EAS [13]. Pediatric EAS-associated hepatic tumors, except benign PHNET, are large (≥10 cm) unlike the hepatic lesion in our patient that was relatively small (~4 cm). The lesion had cystic and calcified areas with arterial phase enhancement that persisted in the delayed phase favoring a diagnosis of hemangioma. Mixed echotexture can be observed in any of the aforementioned lesions, whereas calcification is a common feature of CNSET but rare in PHNET [14]. CNSET and NET demonstrate arterial enhancement with washout in the venous phases [15] whereas the persistent/progressive enhancement in the delayed phase is a typical characteristic of a hemangioma that led to a misdiagnosis in our patient. However, PHNET may rarely (~10%) have continued enhancement in the venous (portal and delayed) phase (type II dynamic enhancement pattern) [16], as noted in our patient.

Misdiagnosis of PHNET on ultrasound as hemangioma is not uncommon. In one series, 10 of 11 patients with PHNET were initially diagnosed as hemangioma [17]. EAS-associated tumors are usually DOTANOC avid but the DOTANOC non-avid nature of the lesion further reduced the probability of it being a NET. Although a previous meta-analysis reported a high sensitivity (81-100%) of the ^68^Ga-somatostatin receptor (SSTR) PET/CT to detect the source of EAS [18], a subsequent analysis reported a very low sensitivity from a single-center experience (1/6) and low sensitivity for covert lesions in the systematic literature review (50%) [19]. SSTR PET/CT sensitivity for PHNET may be even lower due to physiological tracer uptake in the liver, gallbladder, and bowel. Hence, the diagnosis of NET should not be excluded in a non-avid lesion on SSTR PET/CT, particularly in the liver.

As the patient was not adequately responding to medical therapy (ketoconazole), further evaluation to explore the excess ACTH secretion from the liver lesion was considered. A selective venous sampling from common hepatic and right internal jugular veins demonstrated an ACTH gradient of >2 suggesting the lesion as the source of EAS. Peripheral regional venous ACTH sampling has been successfully used previously in a few cases with EAS [20]. This is the first case of PHNET which was successfully evaluated with regional venous ACTH sampling, thus demonstrating its diagnostic utility in such a challenging scenario. Nonetheless, absent somatostatin tracer uptake demanded more evidence to confirm the hepatic lesion as the source of ACTH which was established with a biopsy of the lesion with positive ACTH immunostaining. Indeed, to our knowledge, this is the first case of preoperative confirmation of EAS source by ACTH immunostaining.

The patient had clinical and hormonal remission of CS after excision of the PHNET with disease-free survival of five years. This remission was achieved due to the absence of metastases. Although simple excision may be an option for PHNET, as done in our patient, the majority of the other liver sources of pediatric EAS are large and most often need extensive liver resection. Nonetheless, disease-free survival is good. In the systematic review, except for one EAS patient with a hepatic tumor who died due to sepsis in the perioperative period, all others with benign tumors had disease-free survival until the last follow-up. Nevertheless, close clinical and hormonal monitoring for the recurrence of the disease is essential. With the increasing availability of late-night salivary cortisol, this can a simple and effective tool for the hormonal monitoring of disease recurrence in EAS patients.

## Conclusions

We report a rare case of pediatric EAS due to PHNET with diagnostic dilemmas and demonstrate the utility of novel diagnostic modalities such as documentation of a higher ACTH gradient in the hepatic vein and preoperative positive ACTH immunostaining of the suspected hepatic lesion to resolve the dilemma. In the systematic review, CNSET was the most common hepatic tumor associated with pediatric EAS. The majority of EAS-associated hepatic tumors (CNSET and hepatoblastoma) are large (≥10 cm) and need extensive liver resection, whereas benign PHNETs are relatively smaller (<5 cm) but are misdiagnosed as hemangioma by anatomical imaging. SRS often helps to diagnose PHNET but may rarely miss the diagnosis, as observed in our patient. In such scenarios, the aforementioned novel diagnostic options may be explored to confirm a hepatic lesion as the source of excess ACTH.
